# Not the Root of the Problem—Hair Cortisol and Cortisone Do Not Mediate the Effect of Child Maltreatment on Body Mass Index

**DOI:** 10.3389/fpsyt.2020.00387

**Published:** 2020-05-08

**Authors:** Katharina Pittner, Renate S. M. Buisman, Lisa J. M. van den Berg, Laura H. C. G. Compier-de Block, Marieke S. Tollenaar, Marian J. Bakermans-Kranenburg, Marinus H. van IJzendoorn, Bernet M. Elzinga, Lenneke R. A. Alink

**Affiliations:** ^1^Centre for Forensic Family and Youth Care Studies, Leiden University, Leiden, Netherlands; ^2^Leiden Institute for Brain and Cognition (LIBC), Leiden University, Leiden, Netherlands; ^3^Institute of Medical Psychology, Charité Universitätsmedizin Berlin, Corporate Member of Freie Universität Berlin, Humboldt-Universität zu Berlin, and Berlin Institute of Health (BIH), Berlin, Germany; ^4^Faculty of Behavioural and Movement Sciences, Vrije Universiteit Amsterdam, Amsterdam, Netherlands; ^5^Institute of Psychology, Clinical Psychology Unit, Leiden University, Leiden, Netherlands; ^6^Primary Care Unit, School of Clinical Medicine, University of Cambridge, Cambridge, United Kingdom; ^7^Department of Psychology, Education and Child Studies, Erasmus University Rotterdam, Rotterdam, Netherlands

**Keywords:** child maltreatment, abuse, neglect, hair cortisol, hair cortisone, hypothalamic–pituitary–adrenal axis, body mass index

## Abstract

**Background:**

Experiencing maltreatment during childhood exerts substantial stress on the child and increases the risk for overweight and obesity later in life. The current study tests whether hair cortisol—a measure of chronic stress—and its metabolite cortisone mediate the relation between abuse and neglect on the one hand, and body mass index (BMI) on the other.

**Method:**

The sample consisted of 249 participants aged 8 to 87 years (M = 36.13, SD = 19.33). We collected data on child abuse and neglect using questionnaires, measured cortisol and cortisone concentrations in hair, and BMI. In a structural model, the effects of abuse and neglect on hair cortisol, hair cortisone, and BMI were tested, as well as the covariance between hair cortisol and BMI, and hair cortisone and BMI.

**Results:**

Within the sample, 23% were overweight but not obese and 14% were obese. Higher levels of experienced abuse were related to higher cortisone concentrations in hair (β = 0.24, *p* < .001) and higher BMI (β = 0.17, *p* =.04). Neglect was not related to hair cortisol, hair cortisone, or BMI. Hair cortisol and cortisone did not mediate the association between maltreatment, and BMI. Sensitivity analyses demonstrate the same pattern of results in a subsample of adult participants currently not living with their parents. However, in younger participants who were still living with their parents, the associations between abuse and cortisone (β = 0.14, *p* =.35) and abuse and BMI (β = 0.02, *p* =.92) were no longer significant.

**Conclusion:**

These findings confirm that experiencing abuse is related to higher BMI but suggest that hair cortisol and cortisone are not the mechanism underlying the association between child maltreatment and BMI. This is the first study to show abuse may be associated to elevated concentrations of hair cortisone—evidence of long-term alterations in chronic stress levels. Future research may benefit from exploring the effects of maltreatment on weight gain in longitudinal designs, including measures of other potential mediators such as eating as a coping mechanism, and more direct indicators of metabolic health.

## Introduction

Abuse and neglect are adverse childhood experiences that exert substantial stress on the child, and violate expectations of a safe and stable environment (1, 2). Experiencing abuse or neglect during childhood increases the risk for a number of negative mental (3–5) and physical health outcomes (6–8)—among them an increased risk for overweight and obesity later in life (9–11). The relation between childhood maltreatment and overweight has been confirmed by two meta-analyses including 190,285 and 112,708 participants, respectively (12, 13). Longitudinal studies with several assessments have shown that maltreatment is related to accelerated weight gain (14–17), but report conflicting results with regard to which type of maltreatment drives this effect.

Overweight and obesity present a global health challenge with rising prevalence (18) and with increased risk for diabetes, cardiovascular disease, cancer, and overall mortality (19–24). Unfortunately, it has proven difficult to develop effective interventions to prevent obesity (25), but targeting the mechanisms that underlie the relation between maltreatment and weight gain, such as stress, might offer new effective strategies (26). It has been proposed that mental health mediates the association between maltreatment and BMI. One study investigated posttraumatic stress disorder as a potential mechanism but only found a weak mediation effect (27). Another study found that depression fully mediated the association between physical abuse and BMI in girls but not in boys and did not play a role for other types of maltreatment (17). An alternative approach would be to focus on biological factors such as stress physiology. It has been suggested that maltreatment experiences and increases in BMI could be connected through chronically elevated levels of cortisol (9, 12). Cortisol has been implicated both as a consequence of maltreatment and as a factor involved in weight gain. Therefore, the current study tests the mediational role of hair cortisol (and its metabolite cortisone) in the association between abuse and neglect on the one hand, and BMI on the other hand.

If abuse and neglect are encountered during developmentally sensitive periods, they have the potential to not only elicit an acute stress response but also to reprogram one of the central human stress response systems: the hypothalamic–pituitary–adrenal axis [HPA axis; (28)]. When confronted with a stressor the HPA axis is activated and after a number of intermediary steps, cortisol is released from the adrenal gland and prepares the body to respond to danger (29). Therefore, HPA axis activity is often indexed by measuring cortisol levels. One meta-analysis found evidence of blunted wake-up cortisol levels in maltreated children and adults, with stronger effect sizes in agency-referred samples, but no effect of maltreatment on the cortisol awakening response and the diurnal cortisol pattern (30). Another meta-analysis focused on studies measuring the cortisol response following a social stressor (31). There was evidence of blunted cortisol reactivity in maltreated individuals and this effect was stronger in adults than in children. This can be explained from a theoretical perspective: Both the allostatic load model and the attenuation hypothesis argue that stress will initially elicit an increased stress response in the form of heightened cortisol levels, but over a prolonged period, will result in a blunted stress response (32, 33).

The majority of the above described research has investigated HPA-axis functioning by measuring circulating cortisol in saliva, which offers insight into acute cortisol levels across the day and in response to specific stressors. In recent years, measures have been complemented by assessing cortisol concentrations in hair samples, which represent a more chronic measure of cortisol (34, 35). Cortisol circulating in the bloodstream is incorporated by the hair through passive diffusion.

Several studies have found associations between experienced maltreatment and hair cortisol levels later in life (e.g., 36, 37). A meta-analysis found evidence of elevated cortisol concentrations in hair following trauma sometimes years later (including maltreatment)—albeit with small effect sizes (38). Interestingly, this study revealed two classes of effects. The first class consisted of studies that showed hypocortisolism with a moderate effect size whereas the second class consisted of studies that showed hypercortisolism with a small effect size. While the first class of studies had larger effect sizes, it contained fewer studies: four compared to 24 in the second class. All four studies in the first class investigated child maltreatment. The second class covered a broader spectrum of traumatic experiences but also included 16 studies on child maltreatment. The entire meta-analysis included studies with populations of different ages. However, age did not moderate the effect of trauma on hair cortisol levels. Ultimately, it remained unclear which factors determine whether hair cortisol concentration is blunted or elevated following child maltreatment. In the current study we investigated the unique effects of abuse and neglect on hair cortisol levels in a sample with a wide age range.

In addition to cortisol levels, cortisone levels may be relevant in the context of maltreatment and BMI. Cortisol can be metabolized into the inactive cortisone by 11β-hydroxysteroid dehydrogenase (11B-HSD) type 2 (39). Essentially no research has been conducted to investigate the effects of maltreatment on cortisone. Therefore, the present study also includes a measure of hair cortisone.

Stress and cortisol also play a central role in obesity. Early evidence of the causal role cortisol plays in obesity comes from studies observing weight gain in patients who are administered glucocorticoids (40–42). Additional research suggests that cortisol might be involved in fat accumulation and weight gain (43) by inducing greater food intake (44–46) and disrupting the regulation of fat storage (47). Generally, higher levels of cortisol have been linked to higher BMI (48) but there are also examples of hypocortisolism and higher BMI (49). To date, only a few studies have explicitly explored the relationship between cortisol measured in hair and obesity and most of these studies have relatively small sample sizes. Overall, these studies generally find elevated cortisol concentrations in obese children (50) and adults (51, 52) but not all studies find a relationship (53). Also, it has been suggested that the impact of cortisol on weight not only depends on how much cortisol is secreted but also how it is metabolized. The release of more cortisol may be triggered if more cortisol is transformed to cortisone (54).

The current study adds to the existing research by testing the mediating role of hair cortisol—as an objective measure of chronic stress and HPA-axis functioning—in the relation between retrospectively measured maltreatment and the BMI of children and adults. To our knowledge, only one study has investigated cortisol dysregulation as a mediator between maltreatment and BMI (55). This study found that a flatter cortisol awakening response partially mediated the effect of early adversity on BMI. Since the cortisol awakening response is not correlated to cumulative cortisol secretion measured by hair cortisol it is still unknown whether overall cortisol production measured in hair mediates the relation between maltreatment and BMI. In addition, the current study focused on the unique effects of abuse and neglect on cortisol levels and BMI in line with the theoretical proposition that they represent different types of stressors (56). It has been suggested that cortisone is an alternative, parallel measure of cortisol (35). For a better understanding of HPA axis functioning and corticosteroids in the body (57), we investigated the concentration of both the active cortisol and the inactive cortisone in hair.

We expected that abuse and neglect experienced in childhood would be associated with higher BMI at the time of the study. Further, we expected that abuse and neglect would be related to hair cortisol and cortisone concentrations and that these would in turn be related to BMI. We hypothesize that the effect of maltreatment on BMI would be mediated by elevated hair cortisol and cortisone concentrations. Given mixed results in previous research, we explored whether abuse and neglect had differential effects.

It has been argued that the initial response to experiencing maltreatment is an increased stress reaction, but that over time chronic stress results in down-regulation (30, 32, 58). In this study, we included children and adults. As a result, for some participants experiences of abuse and neglect were potentially acute if they still lived with their parents at the time of the study while it was in the past for the participants who had moved out of their parental home—in some cases decades ago. These two groups might display different HPA axis activity. Therefore, we explored whether the same associations would be observed when performing the analyses for these two groups separately. Following the allostatic load model, elevated cortisol levels would be expected in younger participants who had experienced maltreatment and blunted cortisol levels in adult participants who had experienced maltreatment. Moreover, an association between maltreatment and BMI may only arise in adults.

## Method

### Sample

The sample of the current study was drawn from the Dutch 3G Parenting Study that investigates intergenerational transmission of parenting styles, stress and emotion regulation using a multi-generational design (for details see 59, 60).

For this investigation, we included participants if they agreed to provide a hair sample and had sufficient hair growth resulting in a sample of N = 280. Of these participants, n = 31 were excluded because of corticosteroid use in the previous three months. The final sample of 249 participants came from 60 families with an average of 4.15 family members per family (range: 1 to 18), aged 8 to 87 years (M = 36.13, SD = 19.33).

### Procedure

Participants attended one or two 7-hour lab visits at the Leiden University Medical Center with their nuclear families. During these lab visits, participants completed questionnaires, computer tasks, interviews, and family interaction tasks. In addition, samples of hair, saliva, and buccal cells were collected. All participants signed informed consent. Parents cosigned informed consent if the participant was under the age of 18 years. Also, maltreatment questionnaires of underage participants were inspected after each lab visit. Ethical approval was obtained from the Ethics Committee of the Leiden University Medical Centre (reference number: P11.134).

### Instruments

#### Child Maltreatment

We measured child maltreatment experiences with a combination of two self-report questionnaires: the Parent-Child Conflict Tactics Scales (CTSPC, 61) and the Childhood Trauma Questionnaire (CTQ, 62, 63). Four maltreatment subtypes were assessed: 1) physical abuse (13 items; CTSPC), 2) emotional abuse (five items; CTSPC), 3) physical neglect (four items; CTSPC), and 4) emotional neglect (six items; CTSPC and CTQ). The CTSPC and the CTQ use different rating scales to assess frequencies. In order to be consistent across items, a 5-point scale ranging from (1) never to (5) (almost) always was used for all items. The emotional neglect items from the CTQ were reverse coded.

To measure maltreatment, we used a multi-informant approach in which we combined child- and parent-reports. All participants reported on whether they had experienced maltreatment (i.e., child-report). For the child-report score, participants reported on maternal and paternal behavior separately. Per subtype, we first calculated averages for maltreatment perpetrated by mother and maltreatment perpetrated by father. Then, per subtype, the higher score of mother or father was included in the child-report score. In addition, if a participant participated with a parent, that parent also reported on whether they had perpetrated maltreatment towards that particular participant (i.e., parent-report). The same approach was used to calculate the parent-report score. At least one parent score was available for 61% of the participants. Child-report and parent-report were averaged. Lastly, abuse and neglect scores were calculated by averaging the average for physical and the average for emotional abuse (*r*(247) =.64, *p* < .001) and the average for physical and the average for emotional neglect (*r*(247) =.38, *p* < .001), respectively. Cronbach's αs show acceptable internal consistency for child-report (abuse: α_mother_ =.92, α_father_ =.91; neglect: α_mother_ =.86, α_father_ =.86) and parent-report (abuse: α_mother_ =.78, α_father_ =.84; neglect: α_mother_ =.76, α_father_ =.67).

It should be noted that the procedure for younger participants was slightly adjusted. For participants under 12 years of age, experienced maltreatment was assessed orally and questions about very severe physical abuse were omitted. Participants who were 12 years or older and living with their parents at the time of the study indicated whether they had experienced maltreatment within the last year or in the years before. Per item, the higher score of these two was included. For the full questionnaire and further details, see **Data Sheet 1**.

#### Hair Cortisol and Cortisone

During the lab visit, a research assistant cut approximately 100 hairs closely from the scalp at the back of the head. In most cases, the most proximal 3 cm were analyzed. For six participants, the available hair was shorter than 3 cm: for one participant 1 cm of hair was available, for two participants 2 cm of hair, and for three participants 2.5 cm of hair. Hair samples were prepared by weighing them, cutting them in smaller pieces with surgical scissors, and washing them with 1.0 ml of LC—MS grade isopropanol for 2 min. The hair samples were incubated over night with 1.4 ml LC—MS grade methanol and in presence of 100 µl internal standard (cortisol-d4) for 18 h at 25°C while gently shaking to extract cortisol and cortisone. In line with previous research (e.g., 34, 57), we log-transformed to reduce skewness.

At a growth-rate of approximately one centimeter per month, one centimeter of hair represents the cumulative cortisol secretion of the past month (64). It is possible to reliably assess cortisol in the 3 cm of hair most proximal to the scalp, i.e., representing a retrospective measure of the last three months (35). Hair cortisol has been shown to be related to average salivary cortisol levels from repeated measures across several days but is not associated to the cortisol awakening response or the diurnal cortisol pattern (65–67). Moreover, test-retest correlations indicate that hair cortisol is more stable over time than cortisol measured in saliva or urine (65, 68).

#### BMI

Participants either self-reported on height and weight or, if they did not know, their height and weight were measured by a research assistant. BMI was calculated from these height and weight measures (kilograms/meters^2^). BMI and age were correlated [*r*(233) =.56, *p* < .001]. When assessing BMI in children and adolescents, it has been recommended to use age and gender specific scores based on external reference values (69). Therefore, for participants younger than 21 years, raw BMI scores were transformed into standard deviation scores (SDS) based on a representative Dutch sample (70) applying the LMS method (71) in the software package *childsds* (72) implemented in R (73). For individuals of 21 years and older, no SDS were available. In order to combine scores from both age groups, adult BMI scores were transformed by regressing out age and sex, and standardizing the residuals. This combined score will be denoted as BMI-*z* in the remainder of the manuscript.

#### Hair Related Variables

We assessed several factors that might affect hair cortisol and cortisone levels using a questionnaire. We used open questions to ask participants about their ethnicity, medication use, and hair color. Answers were coded to match previous studies on determinants of hair cortisol and cortisone (34, 57). Ethnicity was recoded into Northern European and other, and medication use into yes or no. Hair color was recoded into black, brown, blond, red, and grey. Further, participants reported whether or not they had dyed, bleached, or permed their hair in the last 3 months, whether they washed their hair more or less than three times a week, whether they had washed their hair more or less than 24 h before taking the hair sample, and whether they had used any hair styling products on the day of sampling. Lastly, the astronomical season of the lab visit was included.

Since most of these confounders, with the exception of season, hair color, and medication, were not related to either hair cortisol or cortisone (see **Table 1**) and adding them increases the number of free parameters, we did not include them in the main analysis but we explored relevant confounders in a sensitivity analysis. All variables were dummy coded. Season was dichotomized to differentiate participants who had participated in summer compared to any other season. Information on hair dying, bleaching, and perming was summarized to indicate whether a participant had undergone any hair treatment in the past three months or not. Adding these confounders as independent variables to be regressed on hair cortisol and cortisone resulted in poor model fit (*X*^2^ = 142.75 (*p* =.00, *df* = 47), RMSEA = 0.90, and CFI = 0.78). Of all the confounding variables, only season (summer vs. other season) was significantly related to cortisol (β = -0.13, *p* =.03) and medication use was related to cortisone (β =.16, p =.02). This is in line with the preliminary analyses (**Table 1**). Contrary to the preliminary analyses, grey hair was neither related to hair cortisol nor to cortisone—likely because the effect was explained by age. Therefore, a sensitivity analysis was performed only including season and medication use as potential confounders.

**Table 1:** Sample characteristics and their association with hair cortisol and cortisone.

Characteristic	Mean(SD)/N(%)	Cortisol M(IQR)	Cortisone M(IQR)
Sex			
Male	77 (31%)	2.01 (2.36)	6.54 (5.50)
Female	172 (69%)	1.93 (2.01)	5.87 (4.80)
Age (in years)	36.13 (19.33)		
Abuse	1.57 (0.42)		
Neglect	1.83 (0.52)		
BMI	23.95 (5.40)		
Medication use			
Yes	98 (39%)	2.37 (2.93)	7.16 (6.61)
No	150 (60%)	1.82 (1.99)*	5.84 (3.77)*
Ethnicity			
Northern European	242 (97%)	1.99 (2.15)	6.10 (4.78)
Other	7 (3%)	2.68 (1.81)	7.90 (6.33)
Hair color			
Black	3 (2%)	1.65 (2.47)	5.18 (11.31)
Brown	70 (28%)	2.18 (2.21)	6.18 (5.55)
Blond (ref)	155 (62%)	1.92 (2.07)	6.06 (4.36)
Red	10 (4%)	1.54 (4.46)	5.54 (16.64)
Grey	10 (4%)	3.10 (8.95)*	10.39 (19.82)*
No hair treatment (last 3 months)	166 (67%)	1.87 (2.11)	6.20 (4.69)
Hair dyed	72 (29%)	2.27 (2.25)	5.87 (5.23)
Hair bleached	33 (13%)	2.91 (2.89)	6.78 (6.48)
Hair permed	5 (2%)	2.69 (0.86)	5.55 (2.55)
Hair washing frequency			
< 3/week	104 (42%)	1.92 (2.56)	5.86 (4.13)
≥3/week	144 (58%)	2.05 (1.84)	6.73 (5.03)
Last hair wash			
≤24 h before sampling	137 (55%)	1.88 (2.20)	6.21 (5.09)
> 24 h before sampling	111 (45%)	2.17 (2.03)	6.16 (4.39)
Use of hair styling products on day of sampling			
Yes	117 (47%)	2.21 (2.20)	6.49 (5.30)
No	130 (52%)	1.83 (1.78)	6.05 (4.38)
Season represented in hair sample			
Winter	49 (20%)	1.81 (2.40)	6.35 (3.21)
Spring	87 (35%)	1.92 (2.20)*	6.05 (5.36)
Summer (ref)	47 (19%)	2.70 (3.09)	6.26 (5.45)
Fall	66 (27%)	1.86 (1.58)*	6.03 (5.32)

Ns may vary due to missing data. For continuous variables, means and standard deviations (SD) are reported. Conversely, for categorical data, Ns and percentages are reported. For categorical data, cortisol and cortisone levels are presented as median (M) and interquartile ranges (IQR) of each group.

#### Demographic Information

Age and sex were included as demographic variables as well as household socioeconomic status (SES). To assess SES we asked participant of 18 years and older about their household income and highest completed education. Yearly household income was measured on a 7-point scale ranging from (1) less than € 15,000 to (7) more than € 65,000. Due to changes in the Dutch educational system, first and second generation participants rated education on a 7-point scale and third generation participants rated education on a 10-point scale. Both scales were rescaled to a 4-point scale. Based on standardized household income and standardized completed educational level a composite household SES score was calculated. If data of two partners living in the same household was available their scores were averaged for the household SES score. Children living with their parents shared their parents' household SES score.

### Analysis

#### Outliers

Outliers (i.e., *z* > ± 3.29) were winsorized (74) by adding or subtracting the difference between the last two acceptable raw values to the last acceptable value. We winsorized two outliers for abuse, one for neglect, four for hair cortisol, seven for hair cortisone, and one for BMI.

#### Missingness

The missing information were as follows: 2.4% hair cortisol, 1.2% hair cortisone, 5.6% BMI, 1.2% hair dyed, 1.2% hair bleached, 0.8% hair permed, 0.4% hair washing frequency, 0.4% on last hair wash, and 0.8% on hair product use. Little's missing completely at random (MCAR) test was not significant (*χ2* = 27.20, *df* = 22, *p* = 0.20) indicating that data was MCAR. Therefore, we imputed missing values using full information maximum likelihood (FIML).

#### Structural Model

The following analyses were implemented in the R package lavaan 0.6-5 (75). A structural model was used to simultaneously test the effects of abuse and neglect on hair cortisol, hair cortisone, BMI-*z*, as well as the covariance between hair cortisol and BMI-*z*, and hair cortisone and BMI-*z*. Theoretically, we expect that glucocorticoids would affect BMI but methodologically we cannot infer causality in our study. By measuring cortisol and cortisone in hair we get an estimate covering the past 3 months, while BMI*-z* was measured during the test day. A model in which glucocorticoids and BMI*-z* mutually reinforce each other is plausible (76, 77). Thus, given the temporal proximity of these measures and the possibility of BMI-*z* affecting HPA-axis functioning, we tested a bidirectional relation rather than a directional effect between cortisol and cortisone on the one hand, and BMI-*z* on the other hand. SES was always included as a covariate. We controlled for age and sex in the prediction of hair cortisol and cortisone but not BMI-*z* because this score was already corrected for age and sex effects. We allowed abuse and neglect to covary with each other and cortisol and cortisone to covary with each other. Based on bivariate correlations, we also included covariation between age and abuse and neglect, as well as SES and neglect.

The model parameters are determined using a maximum likelihood estimator. Standard errors were bootstrapped (1,000 samples) because some of the variables had a non-normal distribution. To assess fit, we inspected comparative fit index (CFI) and the root mean square error of approximation (RMSEA). A CFI above .90 and an RMSEA below .06 was considered to describe a good fit between the model and the observed data (78).

## Results

### Descriptive Statistics

Correlations and descriptive statistics are reported in **Tables 1** and **2**. No differences between men and women were observed but older participants had higher levels of abuse, neglect, cortisol, and cortisone (*p*s < .01). SES was negatively related to neglect and BMI-*z* (*p*s < .05). Abuse and neglect were correlated (*p* < .01) as were cortisol and cortisone (*p* < .01). Lastly, abuse was correlated to cortisol, cortisone, and BMI-*z* (*p* < .05) whereas neglect was only related to cortisone (*p* < .05). For participants who were younger than 21 years, a BMI SDS of 1 or higher was considered overweight and of 2 or higher was considered obese (79). For participants who were 21 and older, the cut-offs were raw BMI scores of 25 and 30 for overweight and obese, respectively (80). Fifty-seven participants (23%) were overweight but not obese and 35 were obese (14%). Note that we use BMI categories only for descriptive purposes here but work with continuous, standardized scores (BMI-*z*) in the analyses for optimal power (81).

**Table 2:** Pearson correlations.

	Abuse	Neglect	Cortisol	Cortisone	BMI-*z*	Sex	Age
Neglect	.54**						
Cortisol	.16*	.12					
Cortisone	.21**	.13*	.71**				
BMI-*z*	.15*	.06	.05	.01			
Sex	.05	.01	-.04	-.12	.01		
Age	.18**	.31**	.33**	.24**	-.06	.09	
SES	-.04	-.16*	.10	.01	-.13*	.01	-.09

Cortisol and cortisone were log-transformed; Sex: male coded as 1, and female coded as 2.

*p < .05; **p < .01.

### Structural Model

**Figure 1:**
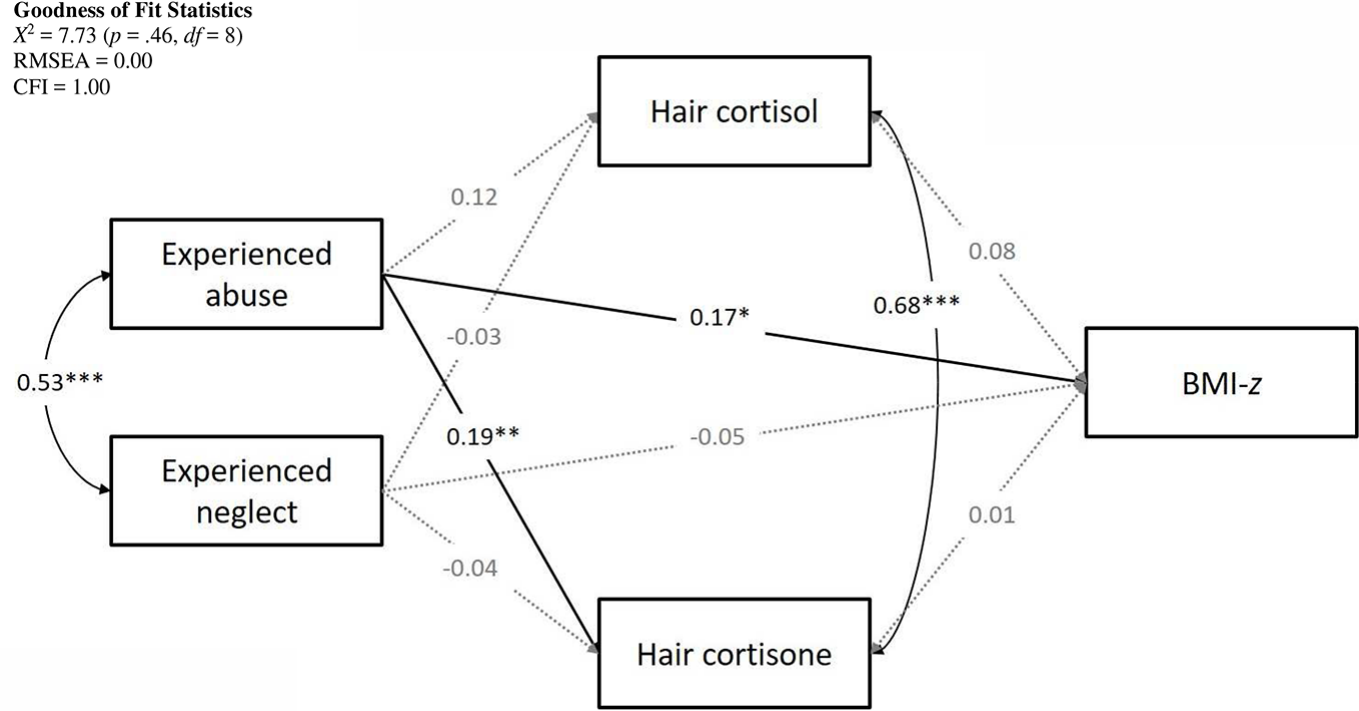
Structural model.

The hypothesized model fit the observed data well with *X*^2^ = 7.73 (*p* =.46, *df* = 8), RMSEA = 0.00, and CFI = 1.00. The complete model with standardized path coefficients is presented in **Figure 1**.

#### Pathways to BMI-z

Lower SES was associated with higher BMI-*z* scores (β = -0.13, *p* =.02). Age and sex were not included as covariates as BMI-*z* was already corrected for age and sex. There was a significant positive association between abuse and BMI-*z* (β = 0.17, *p* =.04) with higher levels of experienced abuse predicting higher BMI-*z*. The effect of experienced neglect on BMI-*z* was not significant (β = -0.05, *p* =.57). Hair cortisol and hair cortisone were not associated with BMI-*z* (cortisol: β = 0.08, *p* =.22; cortisone: β = 0.01, *p* =.85).

#### Pathways to Hair Cortisol

Hair cortisol concentrations were higher in older participants (β = 0.35, *p* < .001) and participants with a higher SES (β = 0.13, *p* =.05) but were not signif ica ntly associated with sex (β = -0.07, *p* =.23). Taking into account these demographic variables, there were no significant relations with abuse (β = 0.12, *p* =.06) or neglect (β = -0.03, *p* =.61).

#### Pathways to Hair Cortisone

We observed higher hair cortisone levels for older participants (β = 0.24, *p* < .001) and men compared to women (β = -.13, *p* =.02). There was no association with SES (β = 0.04, *p* =.16). Controlling for age, sex, and SES, experiencing abuse was significantly related to hair cortisone. Higher levels of experienced abuse were related to greater cortisone concentrations (β = 0.19, *p* < .01). Conversely, the effect of experienced neglect was not significant (β = -0.04, *p* =.56).

#### Indirect Effects

An indirect effect can be significant even if not all of the direct paths are significant. For completeness, we tested whether any of the indirect effects were significant, however, this was not the case (*p*s > .38).

#### Sensitivity and Exploratory Analyses

A number of additional analyses were conducted to exclude alternative explanations for the findings. For one, trauma may affect individuals in a different way depending on whether it is ongoing or not. Therefore, we ran the analyses separately for participants who were still sharing a household with their parents and those who were not. Of the total sample, 173 participants were not living with their parents at the time of the data collection. Running the model in this subsample, we found good model fit [*X*^2^ = 7.61 (*p* =.47, *df* = 8), RMSEA = 0.00, and CFI = 1.00]. We found the same pattern of significant results and similar strength and direction of path coefficients. There were 76 participants who were living with their parents at the time of the data collection. The model fit for this subsample was acceptable [*X*^2^ = 5.79 (*p* =.67, *df* = 8), RMSEA = 0.00, and CFI = 1.00]. However, running the path model for this subgroup led to a different pattern of results with the previously significant effects of abuse on hair cortisone concentration (β = 0.14, *p* =.35) and BMI-*z* (β = 0.02, *p* =.92) disappearing. This might suggest an age effect but could also be the result of a lack of power in the last analysis as this group was smaller than the group of participants who were not living with their parents.

We initially tested the unique effects of cortisol and cortisone. Cortisol and cortisone were highly correlated. In two sensitivity analyses we tested whether cortisone suppressed the effects of cortisol and vice versa by running the analyses for cortisol and cortisone separately. In both cases the estimates of model fit were satisfactory [cortisol only: *X*^2^ = 7.71 (*p* =.46, *df* = 8), RMSEA = 0.00, and CFI = 1.00; cortisone only: *X*^2^ = 7.62 (*p* =.47, *df* = 8), RMSEA = 0.00, and CFI = 1.00] and the same pattern of significant results emerged, with similar path coefficients, suggesting that there was no suppression effect. It has also been suggested that the cortisol:cortisone ratio and the sum of cortisol and cortisone may be meaningful indicators of HPA-axis activity (57). Therefore, we ran two additional models for the ratio and the sum. Both models had good model fit [ratio: *X*^2^ = 7.71 (*p* =.46, *df* = 8), RMSEA = 0.00, and CFI = 1.00; sum: *X*^2^ = 7.64 (*p* =.47, *df* = 8), RMSEA = 0.00, and CFI = 1.00]. Ratio was predicted by age (β =.24, *p* =.001) and SES (β =.14, *p* =.03) but not abuse or neglect. Ratio and BMI-*z* were also not related (β =.10, *p* =.10). The sum of cortisol and cortisone was related to age (β =.32, *p* < .001) and abuse (β =.16, *p* =.02) but not BMI-*z* (β =.03, *p* =.64).

Physical and emotional maltreatment might have differential effects. Therefore, we explored physical and emotional abuse, and emotional neglect in separate models. We omitted physical neglect from these analyses because on its own, physical neglect did not have sufficient variance (M = 1.19, SD = 0.28) or internal reliability (α =.24–.64). For physical abuse, model fit was good [*X*^2^ = 2.16 (*p* =.34, *df* = 2), RMSEA = 0.02, and CFI = 0.99]. Physical abuse showed similar associations with the variables in the model as overall abuse did. There was a significant association with BMI-*z* (β =.15, *p* =.02), cortisol (β =.12, *p* =.05) and cortisone (β =.16, *p* =.01). Results were also similar for emotional abuse [model fit: *X*^2^ = 2.15 (*p* =.34, *df* = 2), RMSEA = 0.02, and CFI = 0.99]. Emotional abuse was associated with BMI-*z* (β =.16, *p* =.02), cortisol (β =.11, *p* =.05), and cortisone (β =.16, *p* =.01). The pattern of results for emotional neglect was also similar to overall neglect. Model fit was adequate [*X*^2^ = 1.91 (*p* =.39, *df* = 2), RMSEA = 0.00, and CFI = 1.00]. Emotional neglect was not associated with any variables of interest (BMI-*z*: *β* =.02, p =.74; cortisol: β =.03, *p* =.58, cortisone: β =.07, *p* =.07).

We also ran the model again with season and medication use as control variables for hair cortisol and cortisone, respectively. This resulted in a model that fit the data adequately [*X*^2^ = 37.86 (*p* =.01, *df* = 20), RMSEA = 0.60, and CFI = 0.95] and did not affect the path coefficients substantially.

## Discussion

This study investigated the effect of experiencing child maltreatment on BMI and the mediating role of chronic stress, indexed by hair cortisol and cortisone. Confirming previous research (12, 13), we found a relation between reported experiences of abuse and higher BMI-*z*. Moreover, we observed higher concentrations of hair cortisone in individuals who had experienced abuse. However, contrary to our expectations, neither hair cortisol nor hair cortisone mediated the association between abuse and BMI-*z*. Neither hair cortisol nor hair cortisone had a direct effect on BMI. Moreover, neglect did not affect hair cortisol, hair cortisone, or BMI-*z*.

In line with most previous research (12, 13), abuse was related to higher BMI. This finding indicates that early experiences can initiate a trajectory that results in weight gain later on and implicates psycho-emotional risk factors in the etiology of overweight. Accordingly, recent models of obesity put greater emphasis on affective aspects of weight gain (76).

As expected, abuse was related to increases in hair cortisone (and trend-level increases in cortisol) suggesting chronic levels of stress in individuals with abuse experiences – in some cases decades after the abuse occurred. This is also in line with the majority of research on maltreatment and hair cortisol which suggest elevated cortisol secretion (38). It is interesting to contrast these findings with evidence of hypocortisolism in maltreated individuals. For instance, meta-analytic evidence suggests that in response to a social stressor, cortisol levels are blunted rather than increased in maltreated individuals. Since hair cortisol represents a cumulative measure of cortisol secretion, this might suggest that abused individuals encounter or perceive more stressful events than non-maltreated individuals—even if their stress response to the particular event is blunted. Alternatively, basal cortisol and cortisone levels may be higher in individuals who experienced childhood abuse.

Evidence of hypocortisolism has generally been interpreted in the context of the allostatic load model: over time the stress response is downregulated to maintain homeostasis. The current study found evidence of elevated cortisone levels in abused individuals—in particular in adults. However, this is not necessarily in contradiction to the allostatic load model since intra-individual cortisone levels may have been downregulated over time, but the cross-sectional nature of the study does not allow us to test this hypothesis. Collectively, these findings show that experiences of abuse get embedded in the body's physiology by reprogramming HPA-axis and higher BMI but that these might be separate processes.

From a methodologic perspective, this finding also supports the measurement of hair cortisone in addition to hair cortisol. At the moment, the majority of studies focus on hair cortisol and do not include a hair cortisone measures. Cortisone levels are generally higher than cortisol levels. It has been suggested that this might make cortisone more readily detectable and a more powerful indicator of chronic stress (57). Effects of abuse on cortisol seem to parallel the effects on cortisone but might require a larger sample size than cortisone. Given that it can be difficult to achieve large sample sizes when studying specific populations, measuring hair cortisone could hold a lot of promise. Moving forward, it will be important to gain a better understanding of the relation between cortisol and cortisone in general and in hair specifically. The correlation between cortisol and cortisone is indicative of a strong relation but also suggests that they are not entirely interchangeable. Functionally, cortisone is less active than cortisol but it is nevertheless an important indicator of HPA axis activity since cortisone and cortisol are closely linked through the 11B-HSD enzymes. Cortisol can be metabolized into cortisone by 11B-HSD type 2 and cortisone can be transformed back into cortisol by 11B-HSD type 1. Therefore, it has been suggested that including measures of 11B-HSD type 1 and 2 activity could provide a completer picture of HPA axis functioning (82). On an endocrine level, 11B-HSD may be involved in the HPA axis feedback loop and contribute to the daily production of cortisol (83).

Our results suggest that the association between childhood abuse and later BMI is not mediated by hair cortisol and cortisone. This can be attributed to the fact that, contrary to our expectations, hair cortisol and cortisone were not associated with BMI-*z*. Previous research has suggested a relation between HPA-axis dysregulation and obesity but results were inconsistent (84). More sensitive measures to assess the relation between HPA-axis functioning and obesity might be site-specific cortisol activity such as intra-adipocyte cortisol concentrations and abdominal adiposity measures to assess body fat distribution (48, 85). Also, differences in hair cortisol may only arise at higher levels of BMI. For instance, one study found that obese participants had higher hair cortisol levels compared to overweight and normal weight participants but no difference between overweight and normal weight participants (52). In the current study, 14% of the sample was obese whereas the majority (63%) was normal weight.

Stress may affect men and women differently. While there is meta-analytic evidence suggesting that the effect of maltreatment on obesity is stronger in women, another meta-analysis suggested that psychosocial stress encountered in adulthood has a stronger effect on BMI in men (86). The same study found stronger effects of psychosocial stress on obesity if the follow-up period was longer. In the current study, two-thirds of the participants were women. Hair cortisol and cortisone measures cover a period of three months whereas years or decades may have elapsed since experiencing abuse. Taken together, these details might explain why we observed an association between abuse and BMI-*z* but no effect of cortisol and cortisone on BMI-*z* because abuse would affect women more strongly and has a longer follow-up period.

Addressing the affective component of obesity could have profound consequences because traditional weight loss interventions primarily focus on non-affective causes by modifying the energy balance by decreasing energy intake and increasing energy expenditure. And while interventions focusing on diet and exercise have been shown to reliably reduce weight, their effects on weight loss are small (87–89). Making matters worse, participants struggle to maintain weight loss and often drop out without completing the intervention (90, 91). For individuals with childhood abuse experiences, these traditional approaches to weight loss may not be sufficient because they do not fully address the underlying problem.

An important issue to consider concerns the timing of interventions. It appears to be generally true that early prevention is more effective than later intervention because smaller changes in behavior are necessary to maintain than to lose weight (92). In the present study, there was no association between abuse and BMI in the younger subgroup who were still living with their parents and are therefore still at risk of parent-to-child abuse. This result should be interpreted with some caution since this subgroup was small. It is interesting to note that in the younger subgroup the association between abuse and cortisone remained similar in strength even though it was not significant anymore. However, the association between abuse and BMI essentially dropped to zero—possibly a sign that this is not solely a power issue. This is in line with meta-analytic evidence showing no effect of maltreatment on BMI in children and adolescents (12). Taken together, this indicates that childhood abuse might be an early risk factor for obesity but the effects may only become apparent later on. Early identification of groups who are at heightened risk for developing overweight is a crucial component of effective prevention.

It may also be relevant to explore other mediating factors such as eating as coping behavior. Research shows that abused individuals are more likely to a develop binge eating disorder (93). This may be an attempt to regulate negative emotions through emotional eating (10, 94). In line with this assumption, symptoms of depression and post-traumatic stress have been found to mediate the relation between maltreatment and obesity—but the evidence is tenuous (17, 27). It has also been argued that unhealthy eating patterns as a result of maltreatment may be associated with decreases in prefrontal cortex volume and less inhibitory control (12). One study has found that sexual abuse resulted in higher BMI in impulsive individuals only. However, the moderating effect of impulsivity was not found for other types of maltreatment (95). More research is needed to explore the mechanisms underlying the effect of maltreatment on BMI.

In the current study, neglect was not related to BMI. The type of maltreatment might affect how the HPA-axis is reprogrammed. It has been argued that abuse and neglect can be differentiated along two dimensions of adverse childhood experiences: abuse representing threat and neglect representing deprivation (96, 97). Threat may not elicit the same stress response as deprivation. For instance, one study found that adolescents who had experienced abuse had chronically elevated stress levels, whereas adolescents who had experienced neglect had blunted stress levels (cited in 98), possibly because threat requires activation of the stress system whereas deprivation does not. Another explanation for this finding might be that participants reported little physical neglect. Therefore, the neglect score primarily represents emotional neglect. Previous evidence suggests that emotional neglect might not be related to higher BMI and obesity (12). It would be interesting to explore how emotional neglect, specifically, differs from the other types of maltreatment. Importantly, these results do not suggest that emotional neglect is not a relevant adverse childhood experience. Rather, these findings suggest that it is time to move away from a purely cumulative model which ignores the type of experience (56). Different types of maltreatment and adverse childhood experiences might affect different developmental pathways in different ways. For instance, emotional neglect might not disrupt stress pathways such as HPA-axis development but it may be detrimental to learning pathways such as reward learning because it deprives the child of positive and sensitive emotional input (56, 99).

### Limitations

The current study had several shortcomings. We did not assess during which developmental period maltreatment occurred. It has been suggested that neglect during the first two to three years of life is particularly crucial in programming HPA-axis functioning (37, 98). Our assessment of maltreatment does not allow us to discern between neglect starting early in the development and neglect that occurred later in the development. Moreover, at least in child reports, any maltreatment that occurred in the first three to four years is unlikely to be remembered and hence, unlikely to be reported (100). Another consequence of not knowing the exact timing of maltreatment experiences is that we could not include the exact time that had elapsed since maltreatment as a potential moderator.

Moreover, the cross-sectional nature of this study means that we cannot draw any conclusion on the causality of the associations. Child maltreatment was assessed retrospectively and thus current stress levels may have affected participants' responses. Generally, agreement between prospective and retrospective measures of child maltreatment tends to be low (101). Baldwin and colleagues (101) argue that prospective and retrospective measures are not interchangeable, but both are valuable. The advantage of retrospective reports is that they may capture more true cases as prospective reports often rely on official records which often underestimate incidence of maltreatment and only capture the most extreme cases. Moreover, cortisone levels are unlikely to account for the association between abuse and cortisone entirely—especially since we used multi-informant scores of maltreatment for most of the participants. What is more, if maltreatment scores were driven by current cortisone levels, we would expect this to affect neglect scores as much as abuse scores which was not the case. Hair cortisol and cortisone levels represent the cumulative secretion of glucocorticoids over the three months prior to measuring BMI. This close temporal proximity makes it difficult to draw strong conclusions with regards to the causal effect of cortisol and cortisone on BMI. For this reason, we tested a bidirectional effect. Since this association was not significant we can conclude that in the present study there was no relation between cortisol and cortisone on the one hand, and BMI on the other hand.

Lastly, BMI is the most commonly used metabolic outcome and is intended to, indirectly, assess body fat percentage (102). However, it is a crude measure and there might be better ways to assess overweight. One disadvantage of BMI is that it is confounded by physical fitness (103, 104). Due to the ease of assessing BMI compared to more direct measures of body fat percentage, it is unlikely to disappear soon. It also allows the comparison with population norms and other studies. For future research, it might be interesting to incorporate other indicators of metabolic health such as leptin deficiency (105) in addition to BMI. It should also be noted, that in some cases, BMI was based on self-report which may introduce some imprecision.

### Conclusion and Implications

Experiencing physical and emotional abuse during childhood can have negative consequences which in some cases are still present decades later. The current study found that experienced abuse was related to elevated levels of hair cortisone and higher BMI. Both elevated cortisone, as a measure of chronic stress, and higher BMI may be related to further negative outcomes and risks such as hypertension, cardiovascular disease, and inflammation (106–110). These findings emphasize that interventions for weight-loss can benefit from integrating psychological components that address stress specifically—especially in individuals with trauma history. Alternatively, finding ways to increase (low to moderate intensity) physical activity might be an accessible way to address both imbalance in the HPA-axis (111) and reduce health risks associated with overweight and obesity (112). The current study found no effect of neglect on cortisol or cortisone or BMI. More and more research is showing differential effects of abuse and neglect (113). This suggests that treatment should be tailored to the specific type of maltreatment individuals have experienced, and we that should not expect that one size fits all.

## Data Availability Statement

The datasets generated for this study are available on request to the corresponding author.

## Ethics Statement

The studies involving human participants were reviewed and approved by Ethics Committee of the Leiden University Medical Centre. Written informed consent to participate in this study was provided by the participants' legal guardian/next of kin.

## Author Contributions

The 3G Parenting Study was conceptualized by MI, BE, and MB-K. KP, LB, RB, and LC-B performed the data collection. KP and LA developed the research question. KP performed the statistical analyses and wrote the manuscript with input from all the authors. All authors approved the final manuscript.

## Funding

The study was supported by the Netherlands Organization for Scientific Research (MB-K: VICI grant [no. 453-09-003]; LA: VIDI grant [no. 016.145.360]; MI: NWO SPINOZA prize) and grants of Leiden University to initiate and support the Research Profile Area Health, Prevention and the Human Life Cycle awarded to MI, P. Assendelft, and B. van Hemert.

## Conflict of Interest

The authors declare that the research was conducted in the absence of any commercial or financial relationships that could be construed as a potential conflict of interest.

## Supplementary Material

Data Sheet 1 Child Maltreatment Instrument.
