# Supplementary material for: Not the Root of the Problem—Hair Cortisol and Cortisone Do Not Mediate the Effect of Child Maltreatment on Body Mass Index
Source: Front Psychiatry. 2020 May 8;11:387. doi: 10.3389/fpsyt.2020.00387 (PMC7225356; doi:10.3389/fpsyt.2020.00387)
Supplement: Data Sheet 1 — Child Maltreatment Instrument. [file DataSheet_1.docx]

**Supplement S1: Child Maltreatment Instrument**

Items 1 through 27 come from the Parent-Child Conflict Tactics Scales (CTSPC; Straus, Hamby, Finkelhor, Moore, & Runyan, 1998). Items 28 through 32 come from the the Childhood Trauma Questionnaire (CTQ; Bernstein et al., 1994; Thombs, Bernstein, Lobbestael, & Arntz, 2009) and were reverse coded. For all items, the response options were: (1) never, (2) once, (3) sometimes, (4) often, (5) (almost) always. Dutch translations were used.

**Child report**

*Children reported on mother and father behavior separately*

1. My father/mother explained to me why something was wrong.^0^
2. My father/mother sent me to the hallway or my room.^0^
3. My father/mother shook me.^1^
4. My father/mother hit me on the bottom with a belt, a hairbrush, a stick, or another hard object.^1^
5. My father/mother gave me something else to do instead of what I was doing wrong.^0^
6. My father/mother yelled at me.^2^
7. My father/mother hit me with a fist or kicked me hard.^1^
8. My father/mother spanked me on the bottom with the bare hand.^1^
9. My father/mother grabbed me around the neck and choked me.^1,*^
10. My father/mother swore or cursed at me.^2^
11. My father/mother beat me up (hit me hard, over and over).^1,*^
12. My father/mother said he/she would send me away or kick me out of the house.^2^
13. My father/mother burned or scolded me on purpose.^1,*^
14. My father/mother threatened to spank or hit me but did not actually do it.^2^
15. My father/mother hit some other part of my body besides the bottom with a belt, a hairbrush, a stick.^1^
16. My father/mother slapped me on the hand, arm, or leg.^1^
17. My father/mother took away privileges or grounded me.^0^
18. My father/mother pinched me.^1^
19. My father/mother threatened me with a knife or a gun.^1,*^
20. My father/mother threw or knocked me down.^1^
21. My father/mother called me dumb or lazy or some other name like that.^2^
22. My father/mother slapped me on the face or head.^1^
23. My father/mother left me home alone, even when some adult should have been with me.^3^
24. My father/mother was so caught up with problems that he/she was not able to show or tell me that he/she loved me.^4^
25. My father/mother was not able to make sure I got the food I needed.^3^
26. My father/mother was not able to make sure I got to a doctor or hospital when I needed it.^3^
27. My father/mother was so drunk or high that he/she had a problem taking care of me.^3^
28. My father/mother helped me feel important and special.^4^
29. My father/mother made me feel loved.^4^
30. My father/mother looked out for me.^4^
31. I felt close to my father/mother.^4^
32. My father/mother was a source of strength.^4^

*Items were omitted in interviews with children younger than 12 years. Note, these items were always included in the parent report, i.e., data from at least one source was available.

**Parent report**

*Parents reported on each child separately.*

1. I explained to [child] why something was wrong.
2. I sent [child] to the hallway or my room.
3. I shook [child].
4. I hit [child] on the bottom with a belt, a hairbrush, a stick, or another hard object.
5. I gave [child] something else to do instead of what I was doing wrong.
6. I yelled at [child].
7. I hit [child] with a fist or kicked me hard.
8. I spanked [child] on the bottom with the bare hand.
9. I grabbed [child] around the neck and choked me.
10. I swore or cursed at [child].
11. I beat [child] up (hit me hard, over and over).
12. I said I would send [child] away or kick [child’s name] out of the house.
13. I burned or scolded [child] on purpose.
14. I threatened to spank or hit [child] but did not actually do it.
15. I hit some other part of [child] body besides the bottom with a belt, a hairbrush, a stick.
16. I slapped [child] on the hand, arm, or leg.
17. I took away privileges or grounded [child].
18. I pinched [child].
19. I threatened [child] with a knife or a gun.
20. I threw or knocked [child] down.
21. I called [child] dumb or lazy or some other name like that.
22. I slapped [child] on the face or head.
23. I left [child] home alone, even when some adult should have been with [child].
24. I was so caught up with my problems that I was not able to show or tell [child] that I loved [child].
25. I was not able to make sure [child] got the food [child] needed.
26. I was not able to make sure [child] got to a doctor or hospital when [child] needed it.
27. I was so drunk or high that I had a problem taking care of [child].
28. I helped [child] feel important and special.
29. I made [child] feel loved.
30. I looked out for [child].
31. [Child] felt close to me.
32. I was a source of strength for [child].

^0^Nonviolent discipline

^1^Physical abuse

^2^Emotional abuse

^3^Physical Neglect

^4^Emotional Neglect
